# Method for Calculating the Simultaneous Maximum Acceptable Risk Threshold (SMART) from Discrete-Choice Experiment Benefit-Risk Studies

**DOI:** 10.1177/0272989X221132266

**Published:** 2022-11-03

**Authors:** Angelyn Otteson Fairchild, Shelby D. Reed, Juan Marcos Gonzalez

**Affiliations:** University of North Carolina at Chapel Hill, Chapel Hill, NC, USA; Duke Clinical Research Institute, Durham, NC, USA; Duke Clinical Research Institute, Durham, NC, USA

**Keywords:** discrete choice experiment, maximum acceptable risk (mar), patient preferences, simultaneous maximum acceptable risk threshold (smart)

## Abstract

**Background:**

Medical decisions require weighing expected benefits of treatment against multiple adverse outcomes under uncertainty (i.e., risks) that must be accepted as a bundle. However, conventional maximum acceptable risk (MAR) estimates derived from discrete-choice experiment benefit-risk studies evaluate the acceptance of individual risks, assuming other risks are fixed, potentially leading decision makers to misinterpret levels of risk acceptance.

**Design:**

Using simulations and a published discrete-choice experiment, we demonstrate a method for identifying multidimensional risk-tolerance measures given a treatment level of benefit.

**Results:**

Simultaneous Maximum Acceptable Risk Thresholds (SMART) represents combinations of risks that would be jointly accepted in exchange for specific treatment benefits. The framework shows how the expectation of utility associated with treatments that involve multiple risks are related even when preferences for potential adverse events are independent. We find that the form of the marginal effects of adverse-event probabilities on the expected utility of treatment determines the magnitude of differences between SMART and conventional single-outcome MAR estimates.

**Limitations:**

Preferences for potential adverse events not considered in a study or preferences for adverse-event attributes held constant in risk-tolerance calculations may affect estimated risk tolerance. Further research is needed to understand the right balance between realistically reflecting clinical treatments with many potential adverse events and the cognitive burden of evaluating risk-risk tradeoffs in research and in practice.

**Conclusions and Implications:**

SMART analysis should be considered in preference studies evaluating the joint acceptance of multiple potential adverse events.

**Highlights:**

- Conventional approaches to calculate maximum-acceptable risk (MAR) using discrete-choice experiment data account for 1 adverse-event risk at a time, requiring that decision makers infer the acceptability of treatments when patients are exposed to multiple risks simultaneously.
- The Simultaneous Maximum Acceptable Risk Threshold (SMART) maps combinations of adverse-event risks that would be jointly acceptable given a specific treatment benefit and provides a transparent and precise portrayal of acceptance of multiple risks.
- Risk levels that would be accepted using individual MAR estimates might not be acceptable when simultaneous risks are considered, especially when marginal expected disutility of risk is decreasing nonlinearly with risk probabilities.
- Preference researchers should calculate SMARTs in any discrete-choice study in which 2 or more adverse-event risks are presented, particularly if risk preferences are nonlinear.

## Introduction

Medical decisions require that decision makers weigh the potential benefits and risks of treatment alternatives. These benefit-risk tradeoffs can be complex, requiring decision makers to understand the clinical outcomes of a therapeutic treatment or medical device and to evaluate how well those outcomes align with treatment goals, preferences, and values. A growing literature aims to quantify patients’ willingness to accept outcomes under uncertainty (i.e., risks) in exchange for specific expected benefits, often based on discrete-choice experiment (DCE) data but increasingly with other methods such as probabilistic threshold technique.^1–5^ Such measures of risk tolerance are increasingly requested and considered by the United States Food and Drug Administration in their approval and clearance processes.^2,6–8^ These studies often report patient preferences framed as the maximum-acceptable risk (MAR) of individual adverse events that stakeholders, typically patients, would be willing to accept in exchange for specific treatment benefits.

The calculations used to estimate MAR evaluate the maximum probability of a single adverse event at which its negative expected utility exactly offsets the utility gain with a defined level of treatment benefit.^1,9,10^ Even when multiple attributes representing adverse-event risks are considered in preference-elicitation tasks, MAR calculations explicitly assume that the probability of only 1 adverse event varies between comparator treatments. If a given treatment carries more than 1 adverse-event risk relative to a comparator, conventional MAR calculations reflect the acceptable probability of each type of adverse event in isolation, thus assuming other risks have zero probability or are at least invariant between treatment options.^i^ With this “all else equal” or “ceteris paribus” assumption, each MAR represents the highest level of acceptable risk in the case in which only 1 risk is different between treatment options. However, decision makers tasked with applying risk-tolerance measures to medical data have no guidance on handling situations in which multiple concurrent risks are relevant and changing simultaneously.

Lacking formal tools to evaluate the acceptance of multiple risks, decision makers must rely on their intuition about acceptable levels of these risks when considered jointly. Although decision makers may be able to infer that tolerance for these risks will be less than the reported acceptable level of the same risk in isolation, individual MARs alone are not sufficient to back-calculate the precise risk tolerance for jointly occurring risks. Without a transparent approach to computing and reporting risk-risk tradeoffs, even well-informed decision makers who understand the full assumptions that underlie MAR estimates may apply individual MAR estimates in ways that overstate, or understate, true risk acceptance in cases in which multiple risks are clinically relevant.

We generalize the conventional single-risk MAR to obtain a simultaneous maximum acceptable risk threshold (SMART), which maps combinations of 2 or more risks that patients would accept in exchange for a given treatment benefit. The SMART framework allows decision makers to transparently evaluate clinical data on treatments that involve multiple simultaneous risks against quantitative estimates of risk tolerance. To our knowledge, a method like this has never been described in the health-preference literature. In this article, we introduce the SMART framework, emphasizing its derivation, construction, and implications, for a methods-oriented audience. A companion article focuses on applications of the SMART framework and is written for decision makers (such as regulators, physicians, or drug developers) who are not primarily methodologists.^11^ In the following sections, we briefly review the background of the MAR, present basic equations used to calculate the conventional MAR, and then derive the SMART framework as a general case. We illustrate computational approaches using hypothetical examples that demonstrate several functional forms. Lastly, we apply the SMART framework to data from a published DCE that included 2 risks.^12^

### Background: MAR

The MAR framework is based on random-utility theory^13^ and expresses the value patients place on improved health outcomes in terms of the probability of an adverse outcome that would offset the value of that benefit. MAR belongs to a class of utility-difference marginal equivalents, the most common being monetary-equivalent value (MEV). While other methods can be used to estimate MARs, DCEs are the most commonly used methods in the elicitation of health-related preferences.^2^ They also provide the most flexible set up to evaluate tolerance for multiple simultaneous risks. Thus, the rest of the discussion will focus on how MARs are derived from DCE data.

The utility-theoretic methods used to calculate MAR estimates from DCEs are described in detail elsewhere.^9,14^ Briefly, respondents in a DCE survey choose among sets of hypothetical treatments characterized by differing levels of features such as health outcomes, elements of the treatment process, and risks of adverse events. Researchers analyze the patterns of choices observed in response to this experimentally controlled variability to statistically identify preference weights that indicate the implicit relative importance of each feature. These preference weights are then used to calculate MARs associated with any probabilistic adverse event included in the study.

In medical contexts, DCEs are used to identify equivalence values for nonmonetary numeraires, including risk, whose disutility exactly offsets the utility of specific improvements in treatment-related health outcomes based on their respective marginal utility.^1^ Using risk as a numeraire introduces the possibility of multiple numeraires with distinct marginal utilities for each risk that may or may not be independent from one another. To resolve this issue and calculate single MAR values, analysts adopt an assumption that only 1 risk can vary in each calculation. Such studies routinely state that respondents would accept up to the MAR of 1 outcome or up to the MAR of another outcome. This assumption is strictly computational and is incorrectly analogous to MEV, in which money is a single fungible numeraire.

However, in clinical decision making, pursuing treatment requires accepting the possibility that 1 or more of the known potential adverse outcomes could occur. While these adverse outcomes may or may not be clinically correlated, or perceived as correlated by patients or other decision makers, the fact remains that patients pursuing treatment are exposed to multiple potential adverse events. Clinicians and patients cannot selectively accept one risk while ignoring other risks that may be associated with an intervention.

This clinical reality is inconsistent with assumptions that underlie MAR calculations. Preference researchers and decision makers who use MAR results to inform their decisions must therefore take steps to bridge the gap between the individual MAR estimates and situations in which multiple risks are relevant. This reality should be correctly incorporated in reporting results from DCEs that inform such decisions, yet the literature does not provide a rigorous, replicable, and precise method to estimate acceptance of multiple risks. The SMART framework that we propose provides such a method. To date, there is also no formal evaluation of the impact that a joint evaluation of risks could have on clinical decisions, and the SMART framework provides a way to makes such assessments.

## Methods

### Calculating the Conventional MAR

Before illustrating how SMART analysis provides a more flexible framework for evaluating multiple risks, we first illustrate how MAR is calculated using conventional methods. Equation (1) represents a basic function that conditions the utility of a potential state that is assumed to be determined by an intervention *i:*

(1) $$U^{i}=f(H^{i},AE_{j})$$

Here, $H^{i}$ represents all features of the intervention *i*, including process features or health outcomes, other than adverse event $AE_{j}$, where *j* indexes the adverse events included in the study. The utility equation is given here in generic form and is flexible enough to accommodate various theoretical or empirical specifications of the relationships among health outcomes, processes, and adverse events.

Assume that preferences for the adverse event and other features of the intervention are separable, such that $f\left(H^{i},AE_{j}\right)=g\left(H^{i}\right)+k(AE_{j})$. This assumption implies that the utility derived from other features of the intervention (including any adverse events other than *J*) is not changed when someone is exposed to the focal adverse event. That is, the ex-ante expected utility of a given treatment profile incorporates the possibility of fatal outcomes or risks that might indicate a change of treatment with a resultant change in benefit. Note that these assumptions do not fundamentally change the derivation of MARs and are covered in Van Houtven et al.^9^

If the incidence of adverse events associated with the intervention is uncertain, the patient can expect to experience $g\left(H^{i}\right)+k(AE_{j})$ with probability $p_{j}^{i}$ and $g\left(H^{i}\right)$ with probability $\left(1-p_{j}^{i}\right)$. The expected utility from intervention *i* can be represented as follows:

(2) $$\begin{array}{c} E\left[U^{i}\right]=p_{j}^{i}\left[g\left(H^{i}\right)+k\left(AE_{j}\right)\right]+\left(1-p_{j}^{i}\right)g\left(H^{i}\right)\\ =g\left(H^{i}\right)+p_{j}^{i}k\left(AE_{j}\right) \end{array}$$

Thus, the expected utility of a given intervention depends on the disutility associated with the adverse event, the probability of that adverse event, and the utility of the other features of the intervention. However, general theories of choice under uncertainty suggest that $p_{j}^{i}$ should be represented as a function,^9,14^ which introduces a weighted probability function *l*(.) that allows for potential for nonlinearity in the marginal effect of risks on expected utility:

(3) $$E\left[U^{i}\right]=\gamma g\left(H\right)+l\left(p_{j}^{i}\right)*k(AE_{j})$$

where $\gamma =l\left(p_{j}^{i}\right)+l\left(1-p_{j}^{i}\right)$ . We consider gamma an adjustment that rescales the expected value of g(H) allowing for the sum of the weighted subjective probabilities to exceed 1 as can be the case with nonexpected utility frameworks.^15–17^ In practice, $\hat{g}(H)=\gamma g(H)$, so we omit it for the rest of the calculations.

MAR is derived by comparing 2 interventions, *i* = [0,1] with differing values for $H^{i}$ and $p_{j}^{i}$, whose expected utilities are represented by equation (3). Consider a pair of interventions in which $g\left(H^{0}\right)<g\left(H^{1}\right)$. The MAR is the difference in the probability of an adverse event associated with these interventions for which $E\left[U^{0}\right]=E\left[U^{1}\right]$. Expanding and rearranging this equation, the following equality defines the MAR:

(4) $$g\left(H^{1}\right)-g\left(H^{0}\right)=\left[l\left(p_{j}^{1}\right)-l\left(p_{j}^{0}\right)\right]*\left[-k(AE_{j})\right]$$

MARs thus can be defined in terms of the maximum absolute level of $p_{j}^{1}$ that would be accepted given $p_{j}^{0}$ and given a generic function for the disutility of risk, as follows:

(5) $$\mathrm{MAR}=p_{j}^{1}=l^{-1}\left[\frac{g\left(H^{1}\right)-g\left(H^{0}\right)}{-k\left(\mathrm{AE}\right)}-l\left(p_{j}^{0}\right)\right]$$

This generic equation can collapse to a simple ratio under certain conditions. If we set $p_{j}^{0}=0$ (i.e., a baseline condition with no additional risk) and assume $l\left(p_{j}^{i}\right)$ is linear in $p_{j}^{i}$, then

(6) $$\mathrm{MAR}=p_{j}^{1}=\frac{g\left(H^{1}\right)-g\left(H^{0}\right)}{-\beta }$$

where β is the marginal effect of the probability of the risk on the treatment expected utility. Note that (5) and (6) are the usual formulations for MARs with single risks. If the baseline probability is greater than zero $\left(p_{j}^{0}>0\right)$, then MARs are added to that baseline risk and represent an increase over the nonzero level of baseline risk.

**Figure 1:**
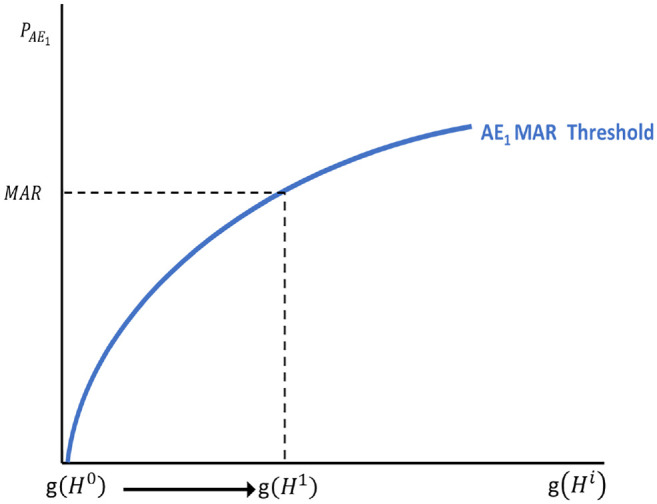
Conventional (single adverse event) maximum-acceptable risk framework.

Figure 1 illustrates the approach used in equation (5) to calculate MAR as a stylized benefit-risk threshold that traces all possible combinations of benefit and risk levels for which given utility gains are exactly offset by the disutility associated with the increased risk. Note that the shape of the threshold depends on the functional form of the weighted probability function, *l*(.). At all points along this threshold, decision makers are indifferent between the baseline low-risk/low-reward profile and the high-risk/high-reward profiles traced by the MAR threshold. Benefit-risk combinations to the right and below the benefit-risk threshold would result in a net positive benefit because the disutility associated with the observed incremental risk for AE_1_ is less than the utility gained from the improved treatment outcome. Benefit/risk combinations above and to the left of the MAR threshold would not be acceptable because the expected disutility associated with risk exceeds the utility associated with the benefit. The dashed lines represent the MAR estimate associated with an improvement from *H*^0^ to a given *H*^1^.

### Interventions Involving Multiple Adverse Event Risks

The flexibility of DCEs facilitates evaluating tradeoffs among a range of beneficial outcomes and multiple probabilistic adverse events. In the previous demonstration of conventional MAR analysis, any probability of adverse events other than the focal $AE_{j}$ used to calculate the MAR was included in the $H^{i}$ vector of treatment processes or health outcomes associated with an intervention, and in practice, their probability of occurrence is assumed to be equal between $H^{0}$ and $H^{1}$. Published DCE studies typically specify a given treatment benefit to vary between interventions, holding all other features, including any additional adverse event risks, as constant between interventions while calculating the MAR for a single adverse event. The process is then repeated for each additional adverse event in turn, holding the probability of all other adverse events at zero or constant between $H^{0}$ and $H^{1}$.

Considering the full conventional MAR for $AE_{1}$ (holding $AE_{2}$ and any subsequent $AE_{j}$ constant) equates the utility-equivalent level of risk to the full utility associated with the intervention; accepting the full single-risk MARs for 2 adverse events would require a doubling of the specified treatment benefit. The benefit of treatment is double counted as a result of the ceteris paribus assumptions embedded in the MAR calculation and exists regardless of whether the adverse events are, in fact, correlated in clinical practice, perceived to be correlated by decision makers, or modeled as correlated in preference research.

Note that the treatment benefit offers a change in total utility, shown as $g\left(H^{1}\right)-g(H^{0})$ in equation (5), which is definitionally net of any disutility associated with the focal AE. For each value of total utility, there is one solution for each risk in the study, representing the conventional single-risk MAR holding any other risks constant across interventions. However, there are an infinite number of combinations of probabilities of any pair of separate risks that could yield an exactly equivalent disutility, and thus, together they define a multidimensional threshold, which we refer to as the Simultaneous Maximum Acceptable Risk Threshold (SMART). This is equivalent to allocating proportions of the available utility to each of the 2 (or more) risks that are present, instead of allowing a single risk to compensate for all of the positive utility generated by the given improvement in health outcomes. Thus, using this method, the utility associated with the treatment benefit is counted only once.

**Figure 2:**
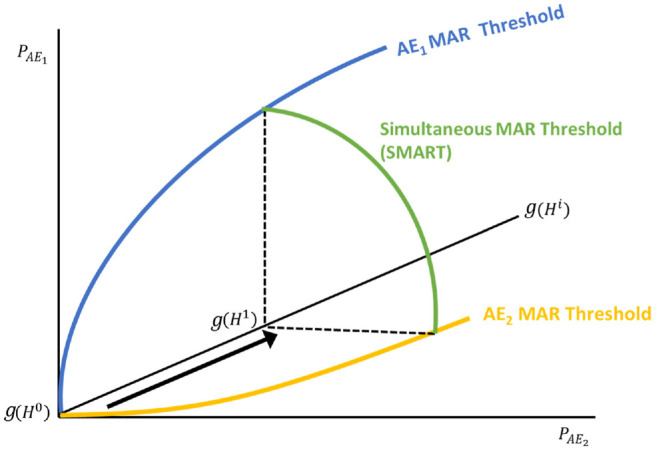
Simultaneous Maximum Acceptable Risk Threshold (SMART).

Figure 2 demonstrates these risk-risk combinations as a stylized 3-dimensional SMART. The figure extends the conventional MAR threshold shown in Figure 1 into a third dimension representing a second adverse event (AE_2_) across various levels of benefit. The green line represents a single benefit increment connecting 2 single MAR thresholds for every benefit increment *g(H^i^)*. The shape of the plane across the full spectrum of treatment benefit *g(H^i^)* depends on respondents’ marginal preferences for avoiding risk, as demonstrated in the next section.

Consider the case in which the utility of intervention *i* is defined by a vector of nonrisk features or outcomes ( $H^{i}$) and by the probabilities of 2 (or more) clinically independent AEs. This case is not aligned with the conventional MAR assumption that each risk occurs in isolation. To represent this situation mathematically, equation (3) can be expanded as follows:

(7) $$E\left[U^{i}\right]=g\left(H^{i}\right)+\sum_{j}l_{j}\left(p_{j}^{i}\right)*k_{j}(AE_{j})$$

where each potential adverse event *j* is included additively in the expected utility function rather than incorporated into $H^{i}$ . The framework can flexibly accommodate 2, 3, or more simultaneous risks. In addition, (4) can be expanded to accommodate the additional adverse events:

(8) $$g\left(H^{1}\right)-g\left(H^{0}\right)=\sum_{j}\left[l_{j}\left(p_{j}^{1}\right)-l_{j}\left(p_{j}^{0}\right)\right]*\left[-k_{j}(AE_{j})\right]$$

The SMART approach defines the set of $p_{j}^{1}$ values for all *j* for which equation (8) holds. Thus, the SMART has the following form:

(9) $$\mathrm{SMART}=p_{j=1}^{1}=l^{-1}\left(\frac{g\left(H^{1}\right)-g\left(H^{0}\right)-\sum_{j=2}^{J}\left[l_{j}\left(p_{j}^{1}\right)-l_{j}\left(p_{j}^{0}\right)\right]*\left[-k_{j}(AE_{j})\right]}{-k_{j=1}(AE_{j})}-p_{j=1}^{0}\right)$$

This framework collapses to the conventional MAR method if we assume that only 1 focal risk can vary across treatments, such that all $l_{j}\left(p_{j}^{2}\right)-l_{j}\left(p_{j}^{1}\right)=0\;for\;J\neq j$ .

## Results

### Demonstration of SMART with Various Marginal Disutilities of Risk

To demonstrate how interpretation of the SMART analysis is affected by different preference patterns, consider a simple example of preference data featuring 2 probabilistic adverse events. While the SMART framework can accommodate 3 or more risks, we use the 2-risk case because it provides a clear conceptual visualization to aid readers in understanding the intuition underlying the SMART framework. For ease in demonstrating the results, assume that each adverse event has probability levels ranging from 0% to 15%, and each adverse event has an identical overall importance of 7.5 (Table 1). Overall importance here is defined as the maximum change in expected utility induced by increases in the probability of adverse events within the ranges considered. Using this framework, we can adjust the marginal disutility of risk within the fixed range of values and examine the impact on the resulting SMARTs. In these examples, risks are assumed to be preferentially independent, so the value that a person places on changing the probability of AE_1_ does not depend on the absolute probability of AE_2_. Risks are also represented as piecewise linear functions. This representation is one way to operationalize the *l*(.) function, which allows risk preferences to take on a number of nonlinear forms.^14^ Assume also that the change in utility of benefit ( $g\left(H^{1}\right)-g\left(H^{0}\right)$ in the prior equations) is normalized to range from 0 to 10.

**Table 1:** Preference Weights for Adverse Events used in Simultaneous Maximum Acceptable Risk Thresholds (SMART) Examples

		Preference Weights under Assumptions of:		
Adverse Event	Probability, %	Constant Marginal Disutility	Increasing Marginal Disutility	Decreasing Marginal Disutility
AE_1_	0	7.5	7.5	7.5
	3	6	7	3.5
	6	4.5	6	1.5
	10	2.5	4	0.5
	15	0	0	0
AE_2_	0	7.5	7.5	7.5
	3	6	7	3.5
	6	4.5	6	1.5
	10	2.5	4	0.5
	15	0	0	0

**Figure 3:**
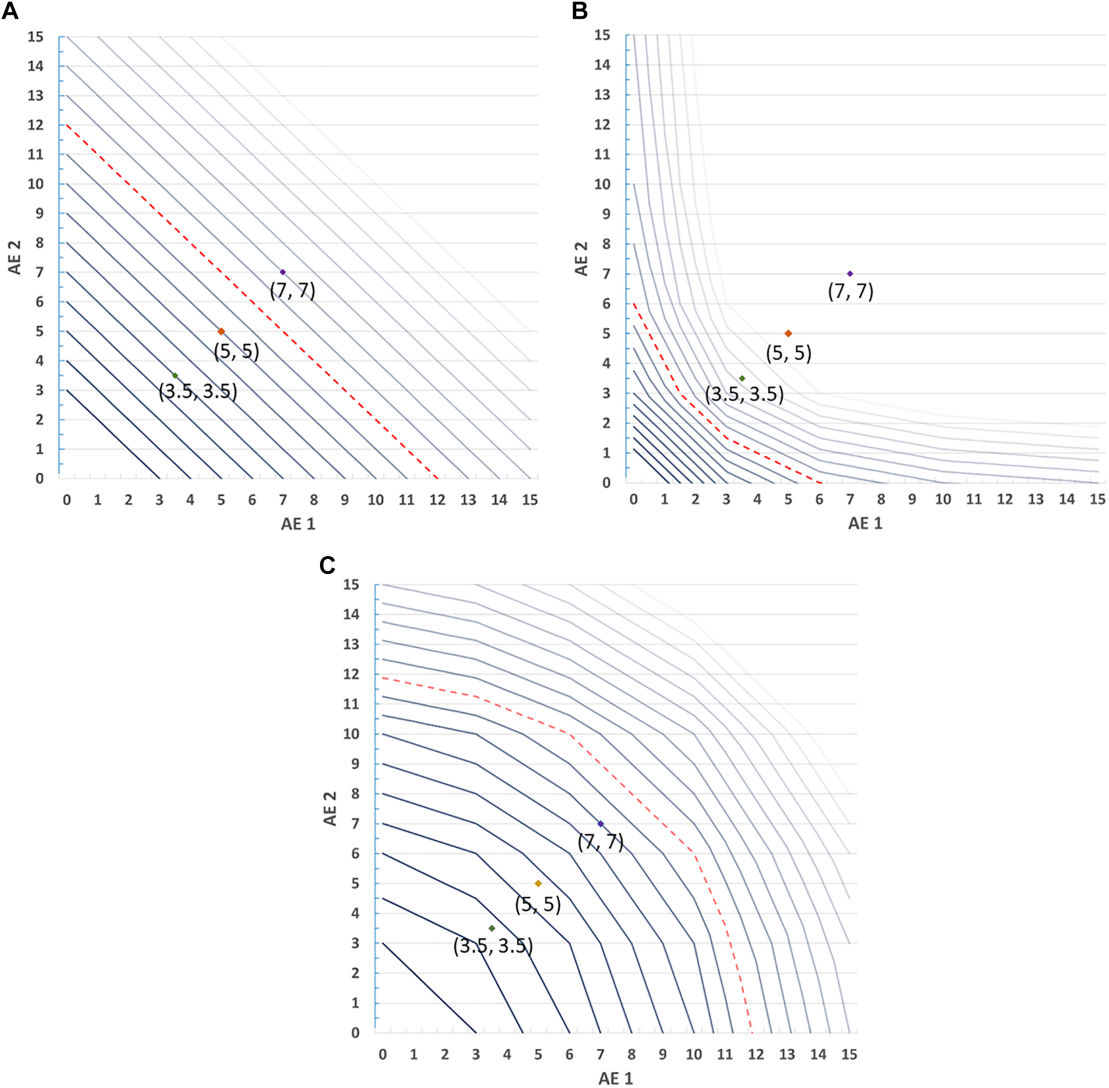
Simultaneous Maximum Acceptable Risk Thresholds (SMART) with varying risk utility functions. (A) Linear. (B) Decreasing marginal disutility. (C) Increasing marginal disutility.

Each of these examples represents different assumptions about the relative value that respondents place on the probability of AE_1_ and AE_2_—in other words, each example assumes a different *l*(.) function. Figure 3 shows the resulting 3-dimensional SMART values for various magnitudes of incremental benefit. Each benefit level is represented by a single line, so that smaller incremental benefits (bolded) result in acceptable risk combinations that fall closer to zero and larger incremental benefits (faded) result in acceptable risk combinations further from zero. The dashed line in each figure highlights a benefit with a utility value or overall importance of 5, for reference. The labeled points at (3.5%, 3.5%), (5%, 5%), and (7.5%, 7.5%) represent pairs of increased probabilities of AE_1_ and AE_2_.

In the case of constant marginal expected disutility of risk (Figure 3a), respondents place equal disutility on each percentage-point increase in probability regardless of its cardinal value. The resulting SMART crest is linear because the rate of exchange between benefits and risks is constant over the range of values in the figure. Although all of the individual risks represented in the labeled points would be accepted under the conventional single-AE MAR calculation when utility equals 5 (the dashed reference line), only the pairings at (3.5%, 3.5%), and (5%, 5%) would be acceptable jointly. The point at (7%, 7%) would not be accepted when compared against the reference utility. Figure 3a represents the simplest scenario in which SMART analysis might be applied—a case with 2 risks that are preference independent and have a linear effect on utility. Because the resulting SMART crest is a linear plane, its slope can be directly calculated for a given change in utility as

$$-\frac{\mathrm{MA}R_{j=1}}{\mathrm{MA}R_{j=2}}$$

This slope represents the rate of exchange (in utility) between the 2 risks, which is constant. Thus, it is possible to calculate any point on the crest by simply evaluating the proportion of the benefit to be offset with each risk. Note that an analogous shortcut calculation is not sufficient to approximate the SMART in any of the other cases, as demonstrated conceptually in Figures 3b and 3c, in which risk preferences are nonlinear, because the exchange rate between risks is not constant.

In the case of diminishing marginal expected disutility of risk (Figure 3b), a single percentage-point increase in risk receives more weight if it is closer to zero than if it is farther from zero. When both risks exhibit diminishing marginal disutility, the SMART crest is a convex surface. Again considering the threshold corresponding to the reference utility of 5, points at (3.5%, 3.5%) and (5%, 5%) would be accepted under conventional MAR analysis but would not be acceptable based on SMART analysis. The risk profile at (7%, 7%) would not be acceptable under either type of analysis.

In the case of increasing marginal expected disutility, a single percentage-point increase in risk receives more value if it is close to 15% and less if it is close to zero (Figure 3c). The resulting SMART crest is a concave surface. In this scenario, all of the plotted reference points would be accepted under either conventional MAR or SMART analysis relative to the reference line.

### Example: DCE with Heart Failure Interventions

Finally, we demonstrate the conventional MAR and SMART approaches using data from a published DCE study, which evaluated patient preferences for heart failure devices. The results of this study are presented in Reed et al.^12^ Briefly, the study evaluated preferences for heart failure devices that could improve physical functioning (benefit) but were associated with increased probability of death within 30 days of implanting the device and increased probability of serious internal bleeding. One additional attribute—hospitalizations in the next 2 y—was also included in the study. Choice data (*n* = 419) were analyzed using a mixed-logit model, and the probabilistic risks of death and bleeding were modeled as independent and categorical effect-coded variables, yielding a piecewise linear *l*(.) function. Table 2 presents the study attributes and the scaled preference weights and 95% confidence intervals associated with each attribute level. Respondents were to suppose that if they did not choose a device alternative, their physical functioning would be equivalent to New York Heart Association (NYHA) class IV and they would be hospitalized 5 times over the following 2 y but would have no additional 30-day risk of death or 30-day risk of severe bleeding.

**Table 2:** Heart Failure Study Attributes, Levels, and Rescaled Preference Weights

	Rescaled Preference Weights (0 ± 10)		
Attributes and levels	Coefficient	Lower CI	Upper CI
Physical functioning equivalent			
NYHA I	10	9.46	10.54
NYHA II	9.28	8.81	9.74
NYHA III	7.75	7.37	8.14
NYHA IV	0.00	−0.95	0.95
Hospitalization in 2 y			
2	0.34	0.08	0.60
3	1.12	0.82	1.41
5	0	−0.25	0.25
30-d risk of death			
1%	0	−0.51	0.51
2%	−0.36	−0.81	0.09
4%	−2.61	−2.98	−2.23
7%	−5.35	−6.02	−4.68
10%	−7.29	−8.06	−6.52
30-d risk of severe bleeding			
1%	0	−0.45	0.45
2%	0.49	0.09	0.89
4%	−1.12	−1.49	−0.76
7%	−2.80	−3.34	−2.25
10%	−3.61	−4.21	−3.00
Device v. no device			
Device	7.28	6.47	8.08
No device	0	−0.81	0.81

Source: Reed et al.^12^

Table 3 replicates the reported individual MAR estimates from this study, indicating the maximum risk of death or the maximum risk of severe internal bleeding that respondents would accept in exchange for the specific levels of improvement in physical functioning as defined by the changes in NYHA class-eq11 symptoms.

**Table 3:** MAR of 30-d Mortality or Severe Bleeding

Improved Outcome		MAR of Death in 30 d,^a^ Mean [95% CI]	MAR of Severe Bleeding,^a^ Mean [95% CI]
From NYHA Class Equivalent	To NYHA Class Equivalent		
IV	III	9.7% [8.2, 13.3]	>20% [0, >20]
	II	12.1% [10.0, 18.4]	>20% [0, >20]
	I	13.2% [10.7, >20]	>20% [0, >20]
III	II	2.0% [1.4, 2.7]	3.7% [2.2, 5.4]
	I	2.7% [2.0, 3.5]	5.0% [3.5, 7.0]
II	I	1.3% [0.2, 2.1]	1.9% [0.2, 3.6]

CI, confidence interval; MAR, maximum-acceptable risk; NYHA, New York Heart Association.

a Baseline risk is 1%. All MAR estimates represent the maximum acceptable increase in risk relative to 1%. MARs were censored at 20%. Source: Reed et al.^12^

**Figure 4:**
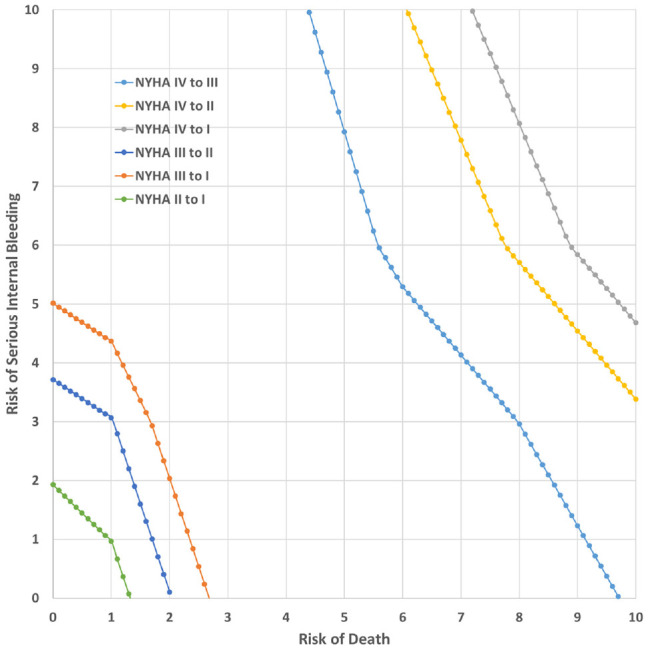
Simultaneous Maximum Acceptable Risk Threshold.

Figure 4 maps the combinations of 30-d risk of death and 30-d risk of severe bleeding that would be acceptable for each of the 6 levels of improvements in physical functioning represented in the study. The X and Y intercepts of each line in Figure 4 are exactly equal to the point estimates for MARs in Table 3 because these intercepts represent the points at which one risk is equal to 1%, the lowest risk level included in the study. The figure demonstrates that, in this case, the multidimensional SMART threshold has a nonuniform shape, which is sometimes convex and sometimes concave depending on the relevant marginal disutility of each change in the level of probabilistic risk modeled as a piecewise function.

## Discussion

By relaxing the restrictive ceteris paribus assumptions that underlie the conventional single-risk MAR framework, the SMART framework enables decision makers to flexibly and systematically evaluate benefit-risk tradeoffs in cases with co-occurring and uncertain adverse events. This is particularly useful in a regulatory context because it can provide decision makers with a more accurate understanding of patients’ preferences for achieving treatment gains in the presence of multiple risks that must be accepted or rejected as a bundle. While the conventional MARs present guideposts for this assessment by representing acceptance of single risks, SMART analysis allows for flexible combinations of multiple risks. As demonstrated (i.e., Figure 3), respondents’ marginal risk preferences can have a considerable impact on the practical interpretation of the SMART versus individual MAR approaches as they relate to the acceptability of clinical risks. In particular, in cases in which risk preferences are linear, SMART analysis provides a transparent estimate of a very simple plane representing risk-risk combinations. For decision makers who understand the tradeoff relationships implied by conventional MARs who might be able to qualitatively or intuitively evaluate joint acceptance of 2 relevant and preference-linear risks, SMART analysis simply adds precision and transparency. For other decision makers who may be less practiced at considering multiple, relevant adverse events when presented with single-risk MARs, transparency afforded by the SMART approach could be enlightening. Furthermore, for cases with nonlinear risk preferences yielding complex and sometimes unexpected joint risk-acceptance thresholds, all types of decision makers may gain considerable value with the SMART approach.

SMART analysis may not be necessary in the simplest case where 1) risk preferences are independent and 2) risk preferences are linear, although it can still be helpful when considering more than 2 risks even when these conditions hold. The proposed SMART analysis provides an efficient means to convey risk tolerance results in any situation in which at least 1 of these conditions is not met. In such cases, it can be very difficult to assess whether a particular combination of risks that are each individually lower than their corresponding conventional MARs would fall below the SMART threshold. Thus, SMART provides a more accurate portrayal of risk acceptance when either risk preferences are not independent or preferences for any risk are nonlinear. The latter condition is particularly important given that nonexpected utility frameworks suggest we could expect nonlinearity in risk preferences.^14,18^

This discussion has bearing on the validity of preference elicitation methods other than DCEs in cases in which multiple risks are relevant. For example, some preference-elicitation methods, such as the probabilistic threshold technique, are designed to evaluate the acceptance of one risk at a time and do not provide information about tolerance of multiple risks simultaneously. However, this implies that the use of these methods is likely most problematic when the marginal expected disutility is not constant. In those situations, deriving MARs at the individual risk levels would not be sufficient to understand the acceptability of treatments with multiple simultaneous risks.

This analysis has focused primarily on the method of calculating and interpreting the SMART. However, it also points to several important implications that merit further research. For example, the analysis raises questions about the appropriate number of probabilistic risks to include or control for in benefit-risk assessments that use DCE methods. DCEs typically include no more than 2 or 3 risk attributes, to avoid presenting respondents with tasks that are too cognitively burdensome.^19,20^ However, our findings suggest that even when preferences for both risks are modeled independently (i.e., they are not interacted with one another directly), the marginal disutility associated with one risk can affect the acceptability of another risk when the ceteris paribus assumption is relaxed. This occurs because the second risk enters the SMART equation as part of the net benefit calculation.

Calculating the SMART also raises important questions about the ways that respondents might interpret and make sense of multiple risks in a treatment profile. In the absence of the typical assumptions used to simplify the MAR calculation, what is the appropriate framework for understanding how respondents think about multiple probabilistic AEs? For example, individuals might simply sum the individual probabilities to estimate an overall chance that an AE will occur, they might consider probabilities as multiplicative, or they might evaluate probabilities influenced by other cognitive biases or simplifying heuristics. Thinking more deeply about risk acceptance in the presence of multiple probabilistic AEs could lead to changes to the standard methods for presenting or modeling risk in DCEs.

In addition, the calculations demonstrated here suggest several important areas for extending the theoretical basis of MAR. For example, the calculations focus on average preference weights similar to those that would be derived by analyzing discrete-choice data using a mixed logit or similar model. If we expand the analysis to consider that preferences for both benefits and risks are heterogeneous across a population, the SMART method could be extended to preferences in latent classes or across a spectrum of preferences. In addition, because this analysis focuses on the conceptual derivation of the SMART threshold taking risk preferences as known, it has not fully incorporated modeling or sampling uncertainty. Future work should demonstrate how the SMART method—including corresponding visualizations—can reflect uncertainty in patients’ acceptance of multiple adverse event risks. In a companion article, we make steps to further these research objectives by exploring regulatory use cases for the SMART method, including the addition of confidence bands along SMART crests, and provide an example with 3 simultaneous risks.^11^

Our discussion here has taken treatment benefits as given. This approach makes the SMART method agnostic to the type of benefit and thus allows for clinical or preference heterogeneity in benefits, so long as the utility of benefit is defined. In addition, DCEs also can include several simultaneous treatment benefits, for example, improvement in several symptom or quality-of-life attributes or reduction in the burden associated with mode of administration. In the MAR calculation, treatment outcomes with one intervention versus another can reflect either a benefit on a single attribute assuming no change in the other attributes or as a composite of the value of improvements across multiple benefit attributes. In regulatory applications, computing the MAR or SMART based on a composite benefit improvement represents the most clinically accurate approach.

Furthermore, some studies model benefits as probabilistic (or, more generally, continuous) and calculate the minimum-acceptable benefit associated with a specific fixed treatment burden. The arguments applied here related to multiple adverse event risks can be similarly applied in a minimum acceptable benefit framework to more precisely reflect the possible combinations of benefits that would be required to exactly offset a given treatment burden (i.e., a specific combination of nonprobabilistic adverse events or other burdensome treatment features).

### Limitations

The SMART method demonstrated here yields the new result that the assumed probability and marginal disutility of risks of nonfocal adverse events influence the acceptability of the focal adverse event, even when there is no direct interaction moderating the relationship between these risks. Logically, this finding extends to risks that cannot be included in a preference elicitation study. It is possible that respondents’ assumptions about the probability of, and value they place on, such risks could influence estimates of risk acceptance. However, the nature of this relationship is not clear. Further research is needed to understand the right balance between realistically reflecting clinical treatments with many potential adverse events and the cognitive burden of evaluating risk-risk tradeoffs in research and in practice. This research should also clarify what assumptions about excluded adverse events, if any, are reasonable. Finally, sophisticated decision makers may be capable of extrapolating single-event MARs in cases in which marginal disutility of both risks are constant. In this case, a SMART analysis yields only a more systematic and transparent representation of tradeoffs between conventional MAR extrema. However, as shown, the SMART analysis is most useful in cases in which the marginal expected disutilities of the relevant risks are increasing or decreasing. Even when constant marginal expected disutilities cannot be ruled out, economic theory suggests broad prevalence of decreasing marginal expected (dis)utility. Thus, sensitivity analysis employing various functional forms consistent with the empirical preference data is advised.

## Conclusion

In cases in which DCE data are used to estimate risk tolerance, SMART methods allow decision makers to precisely consider acceptance of multiple relevant risks—a common clinical reality but contrary to assumptions that underlie conventional MAR calculations. Although the SMART framework is most useful when the marginal disutility of risk is not constant, it also adds value in analyses that feature constant (i.e., linear) risk preferences because it presents a more systematic and transparent mapping to clinical outcomes. More complicated functional forms yield convex or concave SMART thresholds, which further highlight the importance of the method, as our results imply that risks that appear acceptable under the conventional single adverse event MAR framework can be either jointly acceptable or well beyond the jointly acceptable combinations, depending on the marginal disutility of risks. The joint acceptability of risks is critically important in clinical and regulatory decision making and should not be overlooked in discrete choice research.

## Notes

i. A risk could be invariant between alternatives but still affect the tolerability of a second risk differently depending on the numeric probability of risk. Thus, the argument of risk invariance for the consideration of a single adverse event relies on the assumption that risks are preference independent.
